# Cancer Prevention and Treatment on Chinese Social Media: Machine Learning–Based Content Analysis Study

**DOI:** 10.2196/55937

**Published:** 2024-08-14

**Authors:** Keyang Zhao, Xiaojing Li, Jingyang Li

**Affiliations:** 1 School of Media & Communication Shanghai Jiao Tong University Shanghai China; 2 Institute of Psychology and Behavioral Science Shanghai Jiao Tong University Shanghai China; 3 School of Software Shanghai Jiao Tong University Shanghai China

**Keywords:** social media, cancer information, text mining, supervised machine learning, content analysis

## Abstract

**Background:**

Nowadays, social media plays a crucial role in disseminating information about cancer prevention and treatment. A growing body of research has focused on assessing access and communication effects of cancer information on social media. However, there remains a limited understanding of the comprehensive presentation of cancer prevention and treatment methods across social media platforms. Furthermore, research comparing the differences between medical social media (MSM) and common social media (CSM) is also lacking.

**Objective:**

Using big data analytics, this study aims to comprehensively map the characteristics of cancer treatment and prevention information on MSM and CSM. This approach promises to enhance cancer coverage and assist patients in making informed treatment decisions.

**Methods:**

We collected all posts (N=60,843) from 4 medical WeChat official accounts (accounts with professional medical backgrounds, classified as MSM in this paper) and 5 health and lifestyle WeChat official accounts (accounts with nonprofessional medical backgrounds, classified as CSM in this paper). We applied latent Dirichlet allocation topic modeling to extract cancer-related posts (N=8427) and identified 6 cancer themes separately in CSM and MSM. After manually labeling posts according to our codebook, we used a neural-based method for automated labeling. Specifically, we framed our task as a multilabel task and utilized different pretrained models, such as Bidirectional Encoder Representations from Transformers (BERT) and Global Vectors for Word Representation (GloVe), to learn document-level semantic representations for labeling.

**Results:**

We analyzed a total of 4479 articles from MSM and 3948 articles from CSM related to cancer. Among these, 35.52% (2993/8427) contained prevention information and 44.43% (3744/8427) contained treatment information. Themes in CSM were predominantly related to lifestyle, whereas MSM focused more on medical aspects. The most frequently mentioned prevention measures were early screening and testing, healthy diet, and physical exercise. MSM mentioned vaccinations for cancer prevention more frequently compared with CSM. Both types of media provided limited coverage of radiation prevention (including sun protection) and breastfeeding. The most mentioned treatment measures were surgery, chemotherapy, and radiotherapy. Compared with MSM (1137/8427, 13.49%), CSM (2993/8427, 35.52%) focused more on prevention.

**Conclusions:**

The information about cancer prevention and treatment on social media revealed a lack of balance. The focus was primarily limited to a few aspects, indicating a need for broader coverage of prevention measures and treatments in social media. Additionally, the study’s findings underscored the potential of applying machine learning to content analysis as a promising research approach for mapping key dimensions of cancer information on social media. These findings hold methodological and practical significance for future studies and health promotion.

## Introduction

In 2020, 4.57 million new cancer cases were reported in China, accounting for 23.7% of the world’s total [1]. Many of these cancers, however, can be prevented [2,3]. According to the World Health Organization (WHO), 30%-50% of cancers could be avoided through early detection and by reducing exposure to known lifestyle and environmental risks [4]. This underscores the imperative to advance education on cancer prevention and treatment.

Mass media serves not only as a primary channel for disseminating cancer information but also as a potent force in shaping the public health agenda [5,6]. Previous studies have underscored the necessity of understanding how specific cancer-related content is presented in the media. For example, the specific cancer types frequently mentioned in news reports have the potential to influence the public’s perception of the actual incidence of cancer [7].

Nowadays, social media plays an essential role in disseminating health information, coordinating resources, and promoting health campaigns aimed at educating individuals about prevention measures [8]. Additionally, it influences patients’ decision-making processes regarding treatment [9]. A study revealed that social media use correlates with increased awareness of cancer screening in the general population [10]. In recent years, there has been a notable surge in studies evaluating cancer-related content on social media. However, previous studies often focused on specific cancer types [11] and limited aspects of cancer-related issues [12]. The most recent comprehensive systematic content analysis of cancer coverage, conducted in 2013, indicated that cancer news coverage has heavily focused on treatment, while devoting very little attention to prevention, detection, or coping [13].

Evaluating cancer prevention information on social media is crucial for future efforts by health educators and cancer control organizations. Moreover, providing reliable medical information to individuals helps alleviate feelings of fear and uncertainty [14]. Specifically, patients often seek information online when making critical treatment decisions, such as chemotherapy [15]. Therefore, it is significant to comprehensively evaluate the types of treatment information available on social media.

Although many studies have explored cancer-related posts from the perspectives of patients with cancer [16] and caregivers [17], the analysis of posts from medical professionals has been found to be inadequate [18]. This paradox arises from the expectation that medical professionals, given their professional advantages, should take the lead in providing cancer education on social media. Nevertheless, a significant number of studies have highlighted the prevalence of unreliable medical information on social media [19]. A Japanese study highlighted a concerning phenomenon: despite efforts by medical professionals to promote cancer screening online, a significant number of antiscreening activists disseminated contradictory messages on the internet, potentially undermining the effectiveness of cancer education initiatives [20]. Hence, there is an urgent need for the accurate dissemination of health information on social media, with greater involvement from scientists or professional institutions, to combat the spread of misinformation [21]. Despite efforts to study professional medical websites [22] and apps [23], there remains a lack of comprehensive understanding of the content posted on medical social media (MSM). Further study is thus needed to compare the differences between cancer information on social media from professional medical sources and nonprofessional sources to enhance cancer education.

For this study, we defined social media as internet-based platforms characterized by social interactive functions such as reading, commenting, retweeting, and timely interaction [24]. Based on this definition, we further classified 2 types of media based on ownership, content, and contributors: common social media (CSM) and MSM. MSM refers to social media platforms owned by professional medical institutions or organizations. It primarily provides medical and health information by medical professionals, including medical-focused accounts on social media and mobile health apps. CSM refers to social media owned or managed by individuals without medical backgrounds. It mainly provides health and lifestyle content.

Similar to Facebook (Meta Platforms, Inc.), WeChat (Tencent Holdings Limited) is the most popular social media platform in China, installed on more than 90% of smartphones. Zhang et al [25] has indicated that 63.26% of people prefer to obtain health information from WeChat. Unlike other Chinese social media platforms, WeChat has a broader user base that spans various age groups [26]. WeChat Public Accounts (WPAs) operate within the WeChat platform, offering services and information to the public. Many hospitals and primary care institutions in China have increasingly registered WPAs to provide health care services, medical information, health education, and more [27]. Therefore, this study selected WPA as the focus of research.

Based on big data analytics, this study aims to comprehensively map the characteristics of cancer treatment and prevention information on MSM and CSM, which could significantly enhance cancer coverage and assist patients in treatment decision-making. To address the aforementioned research gaps, 2 research questions were formulated.

- Research question 1: What are the characteristics of cancer prevention information discussed on social media? What are the differences between MSM and CSM?
- Research question 2: What are the characteristics of cancer treatment information discussed on social media? What are the differences between MSM and CSM?

## Methods

### Data Collection and Processing

We selected representative WPAs based on the reports from the “Ranking of Influential Health WeChat Public Accounts” [28] and the “2021 National Rankings of Best Hospitals by Specialty” [29]. In this study, we focused on 4 medical WPAs within MSM: Doctor Dingxiang (丁香医生), 91Huayi (华医网), The Cancer Hospital of Chinese Academy of Medical Sciences (中国医学科学院肿瘤医院), and Fudan University Shanghai Cancer Center (复旦大学附属肿瘤医院). We also selected 5 health and lifestyle WeChat Official Accounts classified as CSM for this study: Health Times (健康时报), Family Doctor (家庭医生), CCTV Lifestyle (CCTV 生活圈), Road to Health (健康之路), and Life Times (生命时报).

We implemented a Python-based (Python Foundation) crawler to retrieve posts from the aforementioned WPAs. Subsequently, we implemented a filtration process to eliminate noisy and unreliable data. Note that our focus is on WPAs that provide substantial information, defined as containing no fewer than a certain number of characters. We have deleted documents that contain less than 100 Chinese characters. Furthermore, we have removed figures and videos from the remaining documents. Eventually, we conducted an analysis at the paragraph level. According to our findings from random sampling, noise in articles from WPAs mostly originates from advertisements, which are typically found in specific paragraphs. Therefore, we retained only paragraphs that did not contain advertising keywords. In total, we collected 60,843 posts from these WPAs, comprising 20,654 articles from MSM and 40,189 articles from CSM.

**Figure 1:**
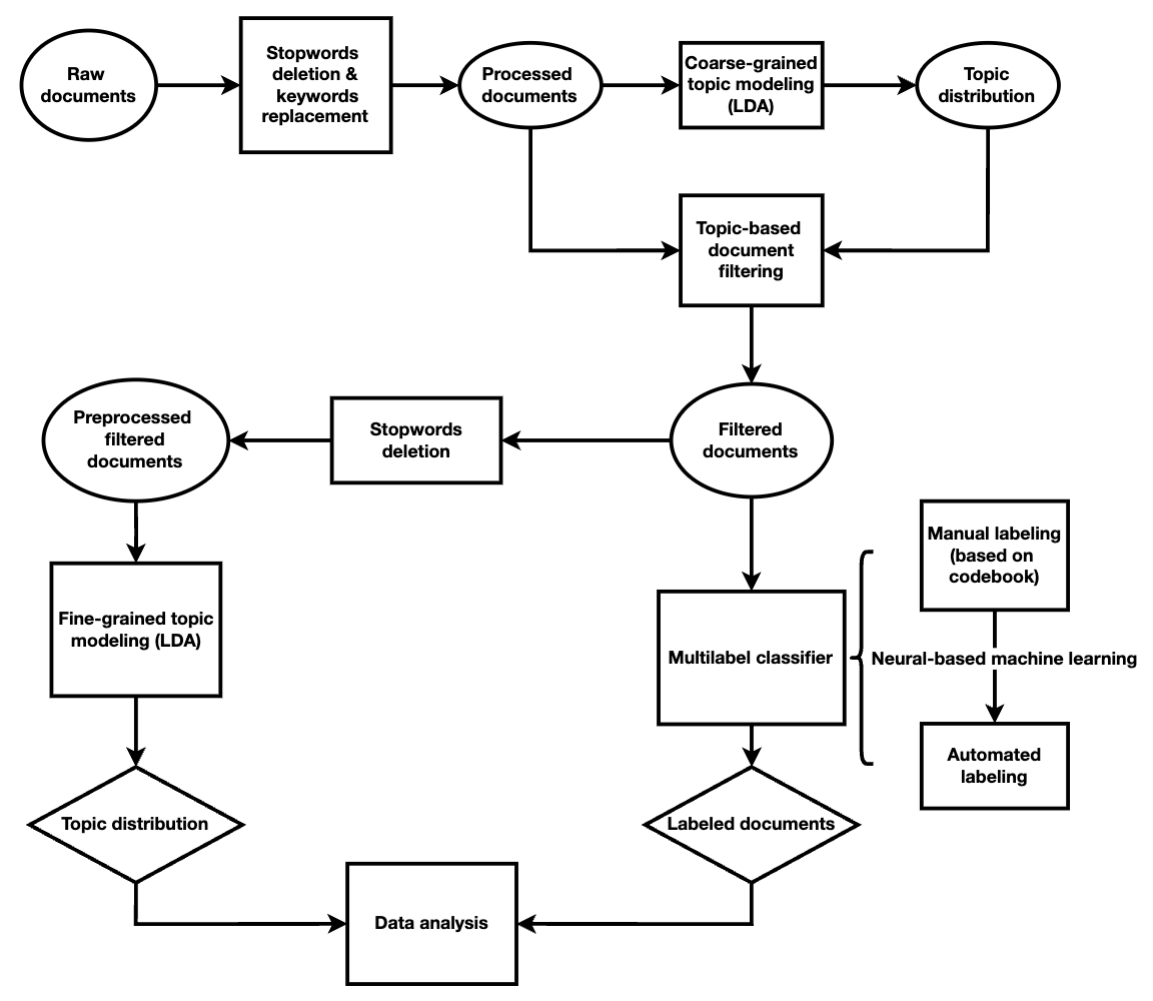
Workflow chart.

The workflow chart in Figure 1 depicts all procedures following data collection and preprocessing. After obtaining meaningful raw documents, we performed word-level segmentation on the texts. We then removed insignificant stopwords and replaced specific types of cancers with a general term to facilitate coarse-grained latent Dirichlet allocation (LDA)–based filtering. Subsequently, we conducted fine-grained LDA topic modeling on the filtered documents without replacing keywords to visualize the topics extracted from the WPAs. Furthermore, we utilized a manually labeled codebook to train a long short-term memory (LSTM) network for document classification into various categories. Finally, we performed data analysis using both the topic distribution derived from fine-grained LDA and the classified documents.

### Latent Dirichlet Allocation Topic Modeling

#### Overview

LDA is a generative statistical model that explains sets of observations by latent groups, revealing why some parts of the data are similar [30]. The LDA algorithm can speculate on the topic distribution of a document.

When comparing LDA with other natural language processing methods such as LSTM-based deep learning, it is worth noting that LDA stands out as an unsupervised learning algorithm. Unlike its counterparts, LDA has the ability to uncover hidden topics without relying on labeled training data. Its strength lies in its capability to automatically identify latent topics within documents by analyzing statistical patterns of word co-occurrences. In addition, LDA provides interpretable outcomes by assigning a probability distribution to each document, representing its association with various topics. Similarly, it assigns a probability distribution to each topic, indicating the prevalence of specific words within that topic. This feature enables researchers to understand the principal themes present in their corpus and the extent to which these themes are manifested in individual documents.

The foundational principle of LDA involves using probabilistic inference to estimate the distribution of topics and word allocations. Specifically, LDA assumes that each document is composed of a mixture of a small number of topics, and each word’s presence can be attributed to one of these topics. This approach allows for overlapping content among documents, rather than strict categorization into separate groups. For a deeper understanding of the technical and theoretical aspects of the LDA algorithm, readers are encouraged to refer to the research conducted by Blei et al [30]. In this context, our primary focus was on the application of the algorithm to our corpus, and the procedure is outlined in the following sections.

#### Document Selection

Initially, document selection involves using a methodological approach to sample documents from the corpus, which may include random selection or be guided by predetermined criteria such as document relevance or popularity within the social media context.

#### Topic Inference

Utilizing LDA or a similar topic modeling technique, we infer the underlying topical structure within each document. This involves modeling documents as mixtures of latent topics represented by a Dirichlet distribution, from which topic proportions are sampled.

#### Topic Assignment to Words

After determining topic proportions, we proceed to assign topics to individual words in the document. Using a multinomial distribution, each word is probabilistically associated with one of the inferred topics based on the previously derived topic proportions.

#### Word Distribution Estimation

Each topic is characterized by a distinct distribution over the vocabulary, representing the likelihood of observing specific words within that topic. Using a Dirichlet distribution, we estimate the word distribution for each inferred topic.

#### Word Generation

Finally, using the multinomial distribution again, we generate words for the document by sampling from the estimated word distribution corresponding to the topic assigned to each word. This iterative process produces synthetic text that mirrors the statistical properties of the original corpus.

To filter out noncancer-related documents in our case, we replaced cancer-related words with “癌症” (cancer or tumor in Chinese) in all documents. We then conducted an LDA analysis to compute the topic distribution of each document and retained documents related to topics where “癌症” appears among the top 10 words.

In our study, we used Python packages such as jieba and gensim for document segmentation and extracting per-topic-per-word probabilities from the model. During segmentation, we applied a stopword dictionary to filter out meaningless words and transformed each document into a cleaned version containing only meaningful words.

During the LDA analysis, to determine the optimal number of topics, our main goal was to compute the topic coherence for various numbers of topics and select the model that yielded the highest coherence score. Coherence measures the interpretability of each topic by assessing whether the words within the same topic are logically associated with each other. The higher the score for a specific number *k*, the more closely related the words are within that topic. In this phase, we used the Python package pyLDAvis to compare coherence scores with different numbers of topics. Subsequently, we filtered and retained only the documents related to cancer topics, resulting in 4479 articles from MSM and 3948 articles from CSM.

Among the filtered articles, we conducted another LDA analysis to extract topics from the original articles without replacing cancer-related words. Using pyLDAvis, we calculated the coherence score and identified 6 topics for both MSM and CSM articles.

**Figure 2:**
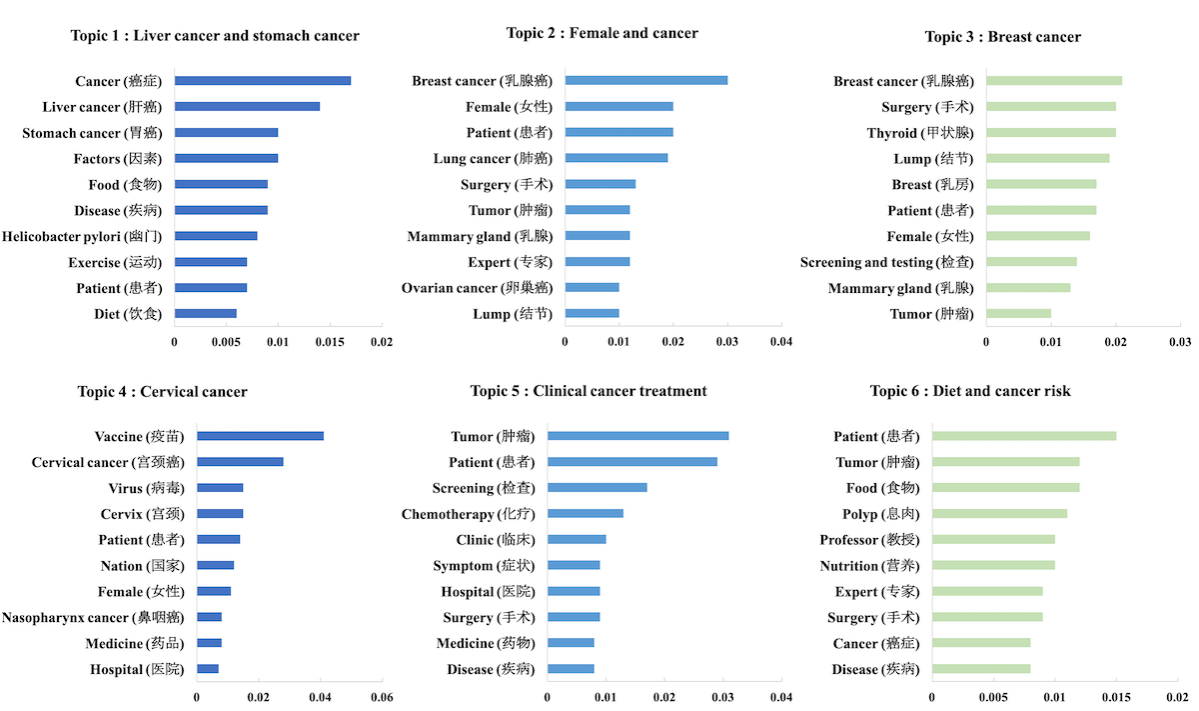
Cancer topics on medical social media (MSM).

**Figure 3:**
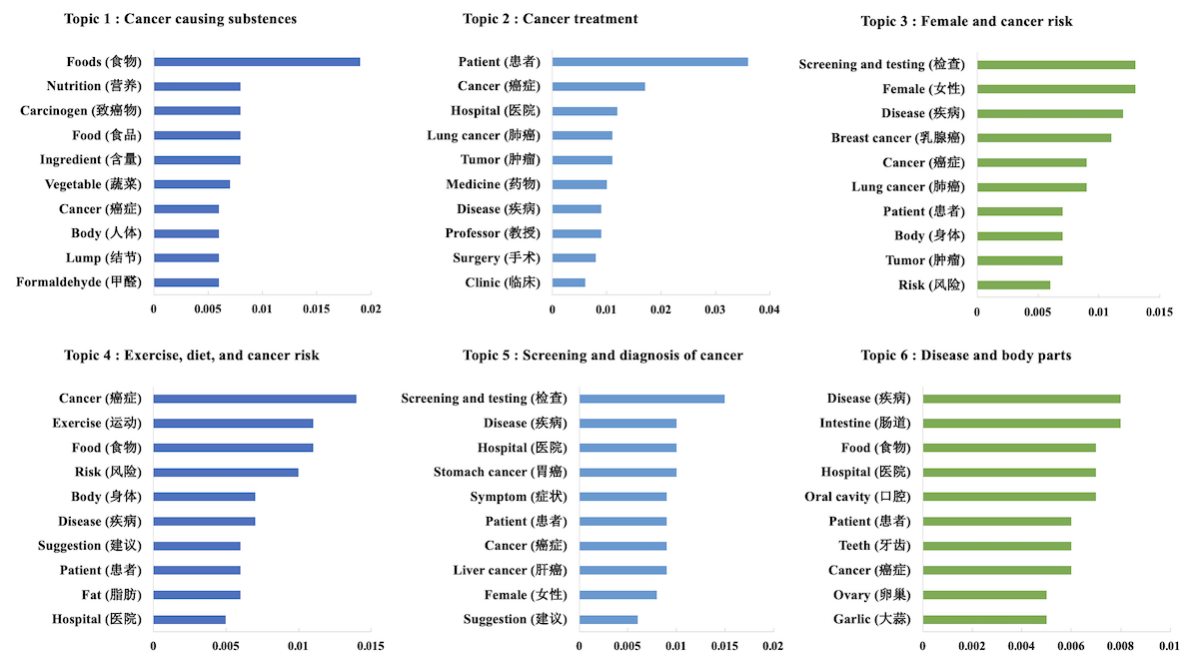
Cancer topics on common social media (CSM).

To visualize the topic modeling results, we created bar graphs where the y-axis indicates the top 10 keywords associated with each topic, and the x-axis represents the weight of each keyword (indicating its contribution to the topic). At the bottom of each graph (Figures 2 and 3), we generalized and presented the name of each topic based on the top 10 most relevant keywords.

### Manual Content Analysis: Coding Procedure

Based on the codebook, 2 independent coders (KZ and JL) engaged in discussions regarding the coding rules to ensure a shared understanding of the conceptual and operational distinctions among the coding items. To ensure the reliability of the coding process, both coders independently coded 100 randomly selected articles. Upon completion of the pilot coding, any disagreements were resolved through discussion between the 2 coders.

For the subsequent coding phase, each coder was assigned an equitable proportion of articles, with 10% of the cancer-related articles randomly sampled from both MSM samples (450/4479) and CSM samples (394/3948). Manual coding was performed on a total of 844 articles, which served as the training data set for the machine learning model. The operational definitions of each coding variable are detailed in Multimedia Appendix 1.

### Coding Measures

#### Cancer Prevention Measures

Coders identified whether an article mentioned any of the following cancer prevention measures [31-35]: (1) avoid tobacco use, (2) maintain a healthy weight, (3) healthy diet, (4) exercise regularly, (5) limit alcohol use, (6) get vaccinated, (7) reduce exposure to ultraviolet radiation and ionizing radiation, (8) avoid urban air pollution and indoor smoke from household use of solid fuels, (9) early screening and detection, (10) breastfeeding, (11) controlling chronic infections, and (12) other prevention measures.

#### Cancer Treatment Measures

Coders identified whether an article mentioned any of the following treatments [36]: (1) surgery (including cryotherapy, lasers, hyperthermia, photodynamic therapy, cuts with scalpels), (2) radiotherapy, (3) chemotherapy, (4) immunotherapy, (5) targeted therapy, (6) hormone therapy, (7) stem cell transplant, (8) precision medicine, (9) cancer biomarker testing, and (10) other treatment measures.

### Neural-Based Machine Learning

In this part, we attempted to label each article using a neural network. As mentioned earlier, we manually labeled 450 MSM articles and 394 CSM articles. We divided the labeled data into a training set and a test set with a ratio of 4:1. We adopted the pretrained Bidirectional Encoder Representations from Transformers (BERT) model. As BERT can only accept inputs with fewer than 512 tokens [37], we segmented each document into pieces of 510 tokens (accounting for BERT’s automatic [CLS] and [SEP] tokens, where [CLS] denotes the start of a sentence or a document, and [SEP] denotes the end of a sentence or a document) with an overlap of 384 tokens between adjacent pieces. We began by utilizing a BERT-based encoder to encode each piece and predict its labels using a multioutput decoder. After predicting labels for each piece, we pooled the outputs for all pieces within the same document and used an LSTM network to predict final labels for each document.

### Ethical Considerations

This study did not require institutional research board review as it did not involve interactions with humans or other living entities, private or personally identifiable information, or any pharmaceuticals or medical devices. The data set consists solely of publicly available social media posts.

## Results

### Cancer Topics on Social Media

Applying LDA, we identified 6 topics each for MSM and CSM articles. The distribution of topics among MSM and CSM is presented in Table 1, while the keyword weights for each topic are illustrated in Figures 2 and 3.

**Table 1:** Distribution of topics on medical social media and common social media (N=8427).a,b

Media type and topic number			Topic description		Articles, n (%)		Top 10 keywords		
**Medical social media**									
	Topic 1	Liver cancer and stomach cancer		1519 (18.03)		Cancer (癌症), liver cancer (肝癌), stomach cancer (胃癌), factors (因素), food (食物), disease (疾病), *Helicobacter pylori* (幽门), exercise (运动), patient (患者), and diet (饮食)			
	Topic 2	Female and cancer		1611 (19.12)		Breast cancer (乳腺癌), female (女性), patient (患者), lung cancer (肺癌), surgery (手术), tumor (肿瘤), mammary gland (乳腺), expert (专家), ovarian cancer (卵巢癌), and lump (结节)			
	Topic 3	Breast cancer		1093 (12.97)		Breast cancer (乳腺癌), surgery (手术), thyroid (甲状腺), lump (结节), breast (乳房), patient (患者), female (女性), screening and testing (检查), mammary gland (乳腺), and tumor (肿瘤)			
	Topic 4	Cervical cancer		1019 (12.09)		Vaccine (疫苗), cervical cancer (宫颈癌), virus (病毒), cervix (宫颈), patient (患者), nation (国家), female (女性), nasopharynx cancer (鼻咽癌), medicine (药品), and hospital (医院)			
	Topic 5	Clinical cancer treatment		2548 (30.24)		Tumor (肿瘤), patient (患者), screening (检查), chemotherapy (化疗), clinic (临床), symptom (症状), hospital (医院), surgery (手术), medicine (药物), and disease (疾病)			
	Topic 6	Diet and cancer risk		1741 (20.66)		Patient (患者), tumor (肿瘤), food (食物), polyp (息肉), professor (教授), nutrition (营养), expert (专家), surgery (手术), cancer (癌症), and disease (疾病)			
**Common social media**									
	Topic 1	Cancer-causing substances		1136 (13.48)		Foods (食物), nutrition (营养), carcinogen (致癌物), food (食品), ingredient (含量), vegetable (蔬菜), cancer (癌症), body (人体), lump (结节), and formaldehyde (甲醛)			
	Topic 2	Cancer treatment		1319 (15.65)		Patient (患者), cancer (癌症), hospital (医院), lung cancer (肺癌), tumor (肿瘤), medicine (药物), disease (疾病), professor (教授), surgery (手术), and clinic (临床)			
	Topic 3	Female and cancer risk		1599 (18.97)		Screening and testing (检查), female (女性), disease (疾病), breast cancer (乳腺癌), cancer (癌症), lung cancer (肺 癌), patient (患者), body (身体), tumor (肿瘤), and risk (风险)			
	Topic 4	Exercise, diet, and cancer risk		1947 (23.10)		Cancer (癌症), exercise (运动), food (食物), risk (风险), body (身体), disease (疾病), suggestion (建议), patient (患者), fat (脂肪), and hospital (医院)			
	Topic 5	Screening and diagnosis of cancer		1790 (21.24)		Screening and testing (检查), disease (疾病), hospital (医院), stomach cancer (胃癌), symptom (症状), patient (患者), cancer (癌症), liver cancer (肝癌), female (女性), and suggestion (建议)			
	Topic 6	Disease and body parts		869 (10.31)		Disease (疾病), intestine (肠道), food (食物), hospital (医院), oral cavity (口腔), patient (患者), teeth (牙齿), cancer (癌症), ovary (卵巢), and garlic (大蒜)			

^a^In each article, different topics may appear at the same time. Therefore, the total frequency of each topic did not equate to the total number of 8427 articles.

^b^To ensure the accuracy of the results, directly translating sampled texts from Chinese into English posed challenges due to differences in semantic elements. In English, cancer screening refers to detecting the possibility of cancer before symptoms appear, while diagnostic tests confirm the presence of cancer after symptoms are observed. However, in Chinese, the term “检查” encompasses both meanings. Therefore, we translated it as both screening and testing.

Among MSM articles, topic 5 was the most frequent (2548/8427, 30.24%), followed by topic 6 (1741/8427, 20.66%) and topic 2 (1611/8427, 19.12%). Both topics 5 and 6 focused on clinical treatments, with topic 5 specifically emphasizing cancer diagnosis. The keywords in topic 6, such as “polyp,” “tumor,” and “surgery,” emphasized the risk and diagnosis of precancerous lesions. Topic 2 primarily focused on cancer surgeries related to breast cancer, lung cancer, and ovarian cancer. The results indicate that MSM articles concentrated on specific cancers with higher incidence in China, including stomach cancer, liver cancer, lung cancer, breast cancer, and cervical cancer [10].

On CSM, topic 4 (1947/8427, 23.10%) had the highest proportion, followed by topic 5 (1790/8427, 21.24%) and topic 3 (1599/8427, 18.97%). Topic 6 had the smallest proportion. Topics 1 and 4 were related to lifestyle. Topic 1 particularly focused on cancer-causing substances, with keywords such as “food,” “nutrition,” and “carcinogen” appearing most frequently. Topic 4 was centered around exercise, diet, and their impact on cancer risk. Topics 3 and 5 were oriented toward cancer screening and diagnosis. Topic 3 specifically focused on female-related cancers, with discussions prominently featuring breast cancer screening and testing. Topic 5 emphasized early detection and diagnosis of stomach and lung cancers, highlighting keywords such as “screening” and “symptom.”

### Cancer Prevention Information

Our experiment on the test set showed that the machine learning model achieved *F*_1_-scores above 85 for both prevention and treatment categories in both MSM and CSM. For subclasses within prevention and treatment, we achieved *F*_1_-scores of at least 70 for dense categories (with an occurrence rate >10%, ie, occurs in >1 of 10 entries) and at least 50 for sparse categories (with an occurrence rate <10%, ie, occurs in <1 of 10 entries). Subsequently, we removed items labeled as “other prevention measures” and “other treatment measures” due to semantic ambiguity.

Table 2 presents the distribution of cancer prevention information across MSM (n=4479) and CSM (n=3948).

**Table 2:** Distribution of cancer prevention information on MSMa and CSMb.

Type of cancer prevention measures	Number of articles on MSM (n=4479), n (%)	Number of articles on CSM (n=3948), n (%)
Articles containing prevention information	1137 (25.39)	1856 (47.01)
Early screening and testing	737 (16.45)	1085 (27.48)
Healthy diet	278 (6.21)	598 (15.15)
Get vaccinated	261 (5.83)	113 (2.86)
Avoid tobacco use	186 (4.15)	368 (9.32)
Exercise regularly	135 (3.01)	661 (16.74)
Limit alcohol use	128 (2.86)	281 (7.12)
Avoid urban air pollution and indoor smoke from household use of solid fuels	19 (0.42)	64 (1.62)
Maintain a healthy weight	18 (0.40)	193 (4.89)
Practice safe sex	12 (0.27)	4 (0.10)
Controlling chronic infections	3 (0.07)	32 (0.81)
Reduce exposure to radiation	2 (0.04)	1 (0.03)
Breastfeeding	1 (0.02)	1 (0.03)

^a^MSM: medical social media.

^b^CSM: common social media.

### Cancer Prevention Information on MSM

The distribution of cancer prevention information on MSM (n=4479) is as follows: articles discussing prevention measures accounted for 25.39% (1137/4479) of all MSM cancer-related articles. The most frequently mentioned measure was “early screening and testing” (737/4479, 16.45%). The second and third most frequently mentioned prevention measures were “healthy diet” (278/4479, 6.21%) and “get vaccinated” (261/4479, 5.83%). The least mentioned prevention measures were “controlling chronic infections” (3/4479, 0.07%), “reduce exposure to radiation” (2/4479, 0.04%), and “breastfeeding” (1/4479, 0.02%), each appearing in only 1-3 articles.

### Cancer Prevention Information on CSM

As many as 1856 out of 3948 (47.01%) articles on CSM referred to cancer prevention information. Among these, “early screening and testing” (1085/3948, 27.48%) was the most commonly mentioned prevention measure. “Exercise regularly” (661/3948, 16.74%) and “healthy diet” (598/3948, 15.15%) were the 2 most frequently mentioned lifestyle-related prevention measures. Additionally, “avoid tobacco use” accounted for 9.32% (368/3948) of mentions. Other lifestyle-related prevention measures were “limit alcohol use” (281/3948, 7.12%) and “maintain a healthy weight” (193/3948, 4.89%). The least mentioned prevention measures were “practice safe sex” (4/3948, 0.10%), “reduce exposure to radiation” (1/3948, 0.03%), and “breastfeeding” (1/3948, 0.03%), each appearing in only 1-4 articles.

### Cancer Prevention Information on Social Media

Table 3 presents the overall distribution of cancer prevention information on social media (N=8427). Notably, CSM showed a stronger focus on cancer prevention (1856/3948, 47.01%) compared with MSM (1137/8427, 13.49%). Both platforms highlighted the importance of early screening and testing. However, MSM placed greater emphasis on vaccination as a prevention measure. In addition to lifestyle-related prevention measures, both CSM and MSM showed relatively less emphasis on avoiding exposure to environmental carcinogens, such as air pollution, indoor smoke, and radiation. “Breastfeeding” was the least mentioned prevention measure (2/3948, 0.05%) on both types of social media.

**Table 3:** Distribution of cancer prevention information on social media.

Type of cancer prevention measures	Number of articles on MSM^a^, n (%)	Number of articles on CSM^b^, n (%)	Number of articles overall (N=8427), n (%)
Articles containing prevention information	1137 (13.49)	1856 (22.02)	2993 (35.52)
Early screening and testing	737 (8.75)	1085 (12.88)	1822 (21.62)
Healthy diet	278 (3.30)	598 (7.10)	876 (10.40)
Get vaccinated	261 (3.10)	113 (1.34)	374 (4.44)
Avoid tobacco use	186 (2.21)	368 (4.37)	554 (6.57)
Exercise regularly	135 (1.60)	661 (7.84)	796 (9.45)
Limit alcohol use	128 (1.52)	281 (3.33)	409 (4.85)
Avoid urban air pollution and indoor smoke from household use of solid fuels	19 (0.23)	64 (0.76)	83 (0.98)
Maintain a healthy weight	18 (0.21)	193 (2.29)	211 (2.50)
Practice safe sex	12 (0.14)	4 (0.05)	16 (0.19)
Controlling chronic infections	3 (0.04)	32 (0.38)	35 (0.42)
Reduce exposure to radiation	2 (0.02)	1 (0.01)	3 (0.04)
Breastfeeding	1 (0.01)	1 (0.01)	2 (0.02)

^a^MSM: medical social media.

^b^CSM: common social media.

### Cancer Treatment Information

Table 4 presents the distribution of cancer treatment information on MSM (n=4479) and CSM (n=3948).

**Table 4:** Distribution of cancer treatment information on MSMa and CSMb.

Type of cancer treatment measures	Number of articles on MSM (n=4479), n (%)	Number of articles on CSM (n=3948), n (%)
Articles containing treatment information	2966 (66.22)	778 (19.71)
Surgery	2045 (45.66)	419 (10.61)
Chemotherapy	1122 (25.05)	285 (7.22)
Radiation therapy	1108 (24.74)	232 (5.88)
Cancer biomarker testing	380 (8.48)	55 (1.39)
Targeted therapy	379 (8.46)	181 (4.58)
Immunotherapy	317 (7.08)	22 (0.56)
Hormone therapy	47 (1.05)	14 (0.35)
Stem cell transplantation therapy	5 (0.11)	0 (0)

^a^MSM: medical social media.

^b^CSM: common social media.

### Cancer Treatment Information on MSM

Cancer treatment information appeared in 66.22% (2966/4479) of MSM posts. “Surgery” was the most frequently mentioned treatment measure (2045/4479, 45.66%), followed by “chemotherapy” (1122/4479, 25.05%) and “radiation therapy” (1108/4479, 24.74%). The proportions of “cancer biomarker testing” (380/4479, 8.48%), “targeted therapy” (379/4479, 8.46%), and “immunotherapy” (317/4479, 7.08%) were comparable. Only a minimal percentage of articles (47/4479, 1.05%) addressed “hormone therapy.” Furthermore, “stem cell transplantation therapy” was mentioned in just 5 out of 4479 (0.11%) articles.

### Cancer Treatment Information on CSM

Cancer treatment information accounted for only 19.71% (778/3948) of CSM posts. “Surgery” was the most frequently mentioned treatment measure (419/3948, 10.61%), followed by “chemotherapy” (285/3948, 7.22%) and “radiation therapy” (232/3948, 5.88%). Relatively, the frequency of “targeted therapy” (181/3948, 4.58%) was similar to that of the first 3 types. However, “cancer biomarker testing” (55/3948, 1.39%), “immunotherapy” (22/3948, 0.56%), and “hormone therapy” (14/3948, 0.35%) appeared rarely on CSM. Notably, there were no articles on CSM mentioning stem cell transplantation.

### Cancer Treatment Information on Social Media

Table 5 shows the overall distribution of cancer treatment information on social media (N=8427). A total of 44.43% (3744/8427) of articles contained treatment information. MSM (2966/8427, 35.20%) discussed treatment information much more frequently than CSM (778/8427, 9.23%). Furthermore, the frequency of all types of treatment measures mentioned was higher on MSM than on CSM. The 3 most frequently mentioned types of treatment measures were surgery (2464/8427, 29.24%), chemotherapy (1407/8427, 16.70%), and radiation therapy (1340/8427, 15.90%). Relatively, MSM (380/8427, 4.51%) showed a higher focus on cancer biomarker testing compared with CSM (55/8427, 0.65%).

**Table 5:** Distribution of cancer treatment information on social media.

Type of cancer treatment measures	Number of articles on MSM^a^, n (%)	Number of articles on CSM^b^, n (%)	Number of articles overall (N=8427), n (%)
Articles containing treatment information	2966 (35.20)	778 (9.23)	3744 (44.43)
Surgery	2045 (24.27)	419 (4.97)	2464 (29.24)
Radiation therapy	1108 (13.15)	232 (2.75)	1340 (15.90)
Chemotherapy	1122 (13.31)	285 (3.38)	1407 (16.70)
Immunotherapy	317 (3.76)	22 (0.26)	339 (4.02)
Targeted therapy	379 (4.50)	181 (2.15)	560 (6.65)
Hormone therapy	47 (0.56)	14 (0.17)	61 (0.72)
Stem cell transplant	5 (0.06)	0 (0.00)	5 (0.06)
Cancer biomarker testing	380 (4.51)	55 (0.65)	435 (5.16)

^a^MSM: medical social media.

^b^CSM: common social media.

## Discussion

### Cancer Topics on MSM and CSM

In MSM, treatment-related topics constituted the largest proportion, featuring keywords related to medical examinations. Conversely, in CSM, the distribution of topics appeared more balanced, with keywords frequently associated with cancer risk and screening. Overall, the distribution of topics on MSM and CSM revealed that CSM placed greater emphasis on lifestyle factors and early screening and testing. Specifically, CSM topics focused more on early cancer screening and addressed cancer types with high incidence rates. By contrast, MSM topics centered more on clinical treatment, medical testing, and the cervical cancer vaccine in cancer prevention. Additionally, MSM focused on types of cancers that are easier to screen and prevent, including liver cancer, stomach cancer, breast cancer, cervical cancer, and colon cancer.

### Cancer Prevention Information on MSM and CSM

Through content analysis, it was found that 35.52% (2993/8427) of articles on social media contained prevention information, and 44.43% (3744/8427) contained treatment information. Compared with MSM (1137/8427, 13.49%), CSM (2993/8427, 35.52%) focused more on prevention.

Primary prevention mainly involves adopting healthy behaviors to lower the risk of developing cancer, which has been proven to have long-term effects on cancer prevention. Secondary prevention focuses on inhibiting or reversing carcinogenesis, including early screening and detection, as well as the treatment or removal of precancerous lesions [38]. Compared with cancer screening and treatment, primary prevention is considered the most cost-effective approach to reducing the cancer burden.

From our results, “early screening and testing” (1822/8427, 21.62%) was the most frequently mentioned prevention measure on both MSM and CSM. According to a cancer study from China, behavioral risk factors were identified as the primary cause of cancer [10]. However, measures related to primary prevention were not frequently mentioned. Additionally, lifestyle-related measures such as “healthy diet,” “regular exercise,” “avoiding tobacco use,” and “limiting alcohol use” were mentioned much less frequently on MSM compared with CSM.

Furthermore, “avoiding tobacco use” (554/8427, 6.57%) and “limiting alcohol use” (409/8427, 4.85%) were rarely mentioned, despite tobacco and alcohol being the leading causes of cancer. In China, public policies on the production, sale, and consumption of alcohol are weaker compared with Western countries. Notably, traditional Chinese customs often promote the belief that moderate drinking is beneficial for health [39]. Moreover, studies indicated that the smoking rate among adult men exceeded 50% in 2015. By 2018, 25.6% of Chinese adults aged 18 and above were smokers, totaling approximately 282 million smokers in China (271 million males and 11 million females) [40]. These statistics align with the consistently high incidence of lung cancer among Chinese men [41]. Simultaneously, the incidence and mortality of lung cancer in Chinese women were more likely associated with exposure to second-hand smoke or occupation-related risk factors.

Although MSM (261/8427, 3.10%) mentioned vaccination more frequently than CSM (113/8427, 1.34%), vaccination was not widely discussed on social media overall (374/8427, 4.44%). The introduction of human papillomavirus vaccination in China has lagged for more than 10 years compared with Western countries. A bivalent vaccine was approved by the Chinese Food and Drug Administration in 2017 but has not been included in the national immunization schedules up to now [42].

According to the “European Code Against Cancer” [43], breastfeeding is recommended as a measure to prevent breast cancer. However, there were no articles mentioning the role of breastfeeding in preventing breast cancer on social media.

One of the least frequently mentioned measures was “radiation protection,” which includes sun protection. Although skin cancer is not as common in China as in Western countries, China has the largest population in the world. A study showed that only 55.2% of Chinese people knew that ultraviolet radiation causes skin cancer [33]. Additional efforts should be made to enhance public awareness of skin cancer prevention through media campaigns.

Overall, our results indicate that social media, especially MSM, focused more on secondary prevention. The outcomes of primary prevention are challenging to identify in individuals, and studies on cancer education may partly explain why primary prevention was often overlooked [44].

### Cancer Treatment Information on MSM and CSM

Compared with a related content analysis study in the United States, our findings also indicate that the media placed greater emphasis on treatment [45]. Treatment information on MSM was more diverse than on CSM, with a higher proportion of the 3 most common cancer treatments—surgery, chemotherapy, and radiation therapy—mentioned on MSM compared with CSM. Notably, CSM (232/8427, 2.75%) mentioned radiation therapy less frequently compared with MSM (1108/8427, 13.15%), despite it being one of the most common cancer treatment measures in clinical practice.

In addition to common treatment methods, other approaches such as targeted therapy (560/8427, 6.65%) and immunotherapy (339/8427, 4.02%) were rarely discussed. This could be attributed to the high costs associated with these treatments. A study revealed that each newly diagnosed patient with cancer in China faced out-of-pocket expenses of US $4947, amounting to 57.5% of the family’s annual income, posing an unaffordable economic burden of 77.6% [46]. In 2017, the Chinese government released the National Health Insurance Coverage (NHIC) policy to improve the accessibility and affordability of innovative anticancer medicines, leading to reduced prices and increased availability and utilization of 15 negotiated drugs. However, a study indicated that the availability of these innovative anticancer drugs remained limited. By 2019, the NHIC policy had benefited 44,600 people, while the number of new cancer cases in China in 2020 was 4.57 million [47]. The promotion of information on innovative therapies helped patients gain a better understanding of their cancer treatment options [48].

### Practical Implications

This research highlighted that MSM did not fully leverage its professional background in providing comprehensive cancer information to the public. In fact, MSM holds substantial potential for contributing to cancer education. The findings from the content analysis also have practical implications for practitioners. They provide valuable insights for experts to assess the effectiveness of social media, monitor the types of information available to the public and patients with cancer, and guide communication and medical professionals in crafting educational and persuasive messages based on widely covered or less attended content.

### Limitations and Future Directions

This study had some limitations. First, we only collected 60,843 articles from 9 WPAs in China. Future research could broaden the scope by collecting data from diverse countries and social media platforms. Second, our manual labeling only extracted 10% (450/4479 for MSM and 394/3948 for CSM) of the samples; the accuracy of the machine learning model could be enhanced by training it with a larger set of labeled articles. Finally, our results only represented the media’s presentation, and the impact of this information on individuals remains unclear. Further work could examine its influence on behavioral intentions or actions related to cancer prevention among the audience.

### Conclusions

The analysis of cancer-related information on social media revealed an imbalance between prevention and treatment content. Overall, there was more treatment information than prevention information. Compared with MSM, CSM mentioned more prevention information. On MSM, the proportion of treatment information was greater than prevention information, whereas on CSM, the 2 were equal. The focus on cancer prevention and treatment information was primarily limited to a few aspects, with a predominant emphasis on secondary prevention rather than primary prevention. There is a need for further improvement in the coverage of prevention measures and treatments for cancer on social media. Additionally, the findings underscored the potential of applying machine learning to content analysis as a promising research paradigm for mapping key dimensions of cancer information on social media. These findings offer methodological and practical significance for future studies and health promotion.

## Appendix

Multimedia Appendix 1 Definitions and descriptions of coding items.

## Abbreviations

BERT: Bidirectional Encoder Representations from Transformers
CSM: common social media
GloVe: Global Vectors for Word Representation
LDA: latent Dirichlet allocation
LSTM: long short-term memory
MSM: medical social media
NHIC: National Health Insurance Coverage
WHO: World Health Organization
WPA: WeChat public account
