# Supplementary material for: Cancer Prevention and Treatment on Chinese Social Media: Machine Learning–Based Content Analysis Study
Source: J Med Internet Res. 2024 Aug 14;26:e55937. doi: 10.2196/55937 (PMC11358654; doi:10.2196/55937)
Supplement: Multimedia Appendix 1 [file jmir_v26i1e55937_app1.pdf]

MULTIMEDIA APPENDIX

Definitions and Descriptions of Coding Items

Coding Item	Definition and descriptions
Cancer Prevention Measures	
Avoid tobacco use	Suggestions related to avoiding tobacco use to prevent cancer (including cigarettes and smokeless tobacco).
Maintain a healthy weight	Suggestions related to maintaining or keeping a healthy weight to prevent cancer.
Healthy diet	Suggestions related to a healthy diet to prevent cancer: eat whole grains, fruit, vegetables, and beans; limit red meat and processed foods; limit fast foods high in fat, starches or sugars.
Exercise regularly	Suggestions related to exercising regularly to prevent cancer, including all types of physical activities.
Limit alcohol use	Suggestions related to limiting alcohol use to prevent cancer.
Practice safe sex	Suggestions related to practicing safe sex to prevent cancer, like using condoms when having sex.
Get vaccinated	Suggestions related to getting vaccinated against hepatitis B and human papillomavirus (HPV) to prevent cancer.
Reduce exposure to ultraviolet radiation and ionizing radiation	Suggestions related to reducing exposure to ultraviolet radiation and ionizing radiation.<br>1. Ultraviolet radiation from sunlight.<br>2. Ionizing radiation includes medical radiation from tests to diagnose cancer such as x-rays, CT scans, fluoroscopy, and nuclear medicine scans. Radon gas in our homes.
Avoid urban air pollution and indoor smoke from household use of solid fuels	Suggestions related to avoiding urban air pollution and indoor smoke from household use of solid fuels to prevent cancer.
Early screening and testing	Suggestions related to early screening and testing to prevent cancer.
Breastfeeding	Suggestions related to breastfeeding to prevent breast cancer (female only).
Controlling chronic infections	Suggestions related to controlling chronic infections to prevent cancer.
Other	Preventive measures do not belong to any of the categories above.
Cancer Treatment Measures	

Surgery	Surgery, when used to treat cancer, is a procedure in which a surgeon removes cancer from the patient's body, including cuts with scalpels, cryotherapy, lasers, hyperthermia, photodynamic therapy.
Radiation therapy	Radiation therapy (also called radiotherapy) is a cancer treatment that uses high doses of radiation to kill cancer cells and shrink tumors.
Chemotherapy	Chemotherapy (also called chemo) is a type of cancer treatment that uses drugs to kill cancer cells, including external beam radiation therapy, brachytherapy, radioactive iodine, I-131.
Immunotherapy	Immunotherapy is a type of cancer treatment that helps your immune system fight cancer. Immunotherapy is a type of biological therapy, including immune checkpoint inhibitors, T-cell transfer therapy, monoclonal antibodies, immune system modulators, etc.
Targeted Therapy	Most targeted therapies are either small-molecule drugs or monoclonal antibodies.
Hormone Therapy	Hormone therapy is a cancer treatment that slows or stops the growth of cancer that uses hormones to grow. Hormone therapy is also called hormonal therapy, hormone treatment, or endocrine therapy.
Stem cell transplant	Stem cell transplants are procedures that restore blood-forming stem cells in people who have had theirs destroyed by the very high doses of chemotherapy or radiation therapy that are used to treat certain cancers.
Cancer biomarker testing	Biomarker testing is a way to look for genes, proteins, and other substances (called biomarkers or tumor markers) that can provide information about cancer. Biomarker testing for cancer treatment may also be called:<br>1. Tumor testing<br>2. Tumor genetic testing<br>3. Genomic testing or genomic profiling<br>4. Molecular testing or molecular profiling<br>5. Somatic testing<br>6. Tumor subtyping.
Other	Treatment measures do not belong to any of the categories above.
